# Incidence of heavy menstrual bleeding and prevalence of co-occurring conditions from seven countries across Europe and North America: an EHDEN real-world data study

**DOI:** 10.1016/j.eclinm.2026.104297

**Published:** 2026-11-03

**Authors:** Marta Pineda-Moncusi, Zsolt Bagyura, Carina Dinkel-Keuthage, Kristina Gemzell-Danielsson, Saeed Hayati, Oskari Heikinheimo, Ronald Herrera, Bruno Imthurn, Sara Khalid, Loretta Zsuzsa Kiss, Angela Leis, Katrin Manlik, Miguel-Angel Mayer, Carsten Möller, Juan Manuel Ramírez Anguita, Nhung T.H. Trinh, Siir Su Saydam, Mariana Schiffer Acar, Hedvig Nordeng, George Argyriou, Katia Verhamme, Gianmario Candore

**Affiliations:** a Health Data Sciences, Centre for Statistics in Medicine, Nuffield Department of Orthopaedics, Rheumatology and Musculoskeletal Sciences (NDORMS), University of Oxford, Oxford, UK; b Semmelweis University, Budapest, Hungary; c Bayer AG, Berlin, Germany; d Department of Women's and Children's Health, Karolinska Institutet, and Karolinska University Hospital, Stockholm, Sweden; e Pharmacoepidemiology and Drug Safety Research Group, Department of Pharmacy, Faculty of Mathematics and Natural Sciences, University of Oslo, Oslo, Norway; f Department of Obstetrics and Gynecology, University of Helsinki and Helsinki University Hospital, Helsinki, Finland; g Boehringer-Ingelheim GmbH, Berlin, Germany; h University of Zurich, Zurich, Switzerland; i Research Programme on Biomedical Informatics, Hospital del Mar and Hospital del Mar Research Institute, Barcelona, Spain; j Bayer CC AG, Basel, Switzerland; k EPAM Systems Inc., RWE, Newtown, USA; l Erasmus University, Rotterdam, Netherlands

**Keywords:** Heavy menstrual bleeding, Federated network study, OMOP CDM, Real-world evidence, Real-world data, EHDEN

## Abstract

**Background:**

Heavy menstrual bleeding (HMB) presents a significant burden for women but is often under-diagnosed and its consequences under-recognised. Improved real-world characterisation of HMB may help identify unmet needs and support earlier and more accurate diagnosis. This study aimed to estimate HMB incidence and prevalence of co-occurring conditions.

**Methods:**

EHDEN HMB was an observational cohort study of nine real-world data sources from seven countries across Europe and North America covering different healthcare settings (primary care-only, hospital-only, primary/specialist non-hospital, all). The study period was from Jan 1, 2000 (or the earliest available date thereafter) to the end of data availability (2021–2024 depending on the data source). Overall and annual incidence rates (IRs) of HMB, as well as the prevalence of co-occurring conditions during the year pre- and post-index (i.e., first HMB diagnosis), were assessed among women aged 11–55 years.

**Findings:**

A total of 3,797,918 women with HMB were included. Overall IR ranged from 0.7 to 25.9 per 1000 person-years, with highest rates observed in data sources encompassing all healthcare settings and lowest in primary care- or hospital-only data. IRs were also highest in women aged 30–44. Pain, anaemia, and dysmenorrhoea were the most common co-occurring conditions both pre-index and post-index (ranging from 5.5% to 21.0% pre-index vs 3.0% to 8.9% post-index for general pain, 3.2% to 20.9% vs 2.0% to 7.0% for narrow/gynaecological pain, 3.4% to 11.5% vs 1.9% to 5.7% for anaemia, and 0.7% to 13.6% vs 0.5% to 5.5% for dysmenorrhoea). Uterine leiomyoma, a diagnosed condition considered to underlie HMB, was also common both pre-index (0.9% to 10.5%) and post-index (1.1% to 9.1%).

**Interpretation:**

To our knowledge, this was the first large-scale real-world study characterising HMB. IR and prevalence of co-occurring conditions varied substantially across healthcare settings and data sources. Changes in prevalence of co-occurring conditions pre- and post-index may reflect changes in clinical management following an HMB diagnosis. Greater awareness of HMB and related conditions may support improved screening and earlier diagnoses, ultimately improving patient outcomes and quality of life.

**Funding:**

Innovative Medicines Initiative 2 Joint Undertaking (no. 806968) with support from European Union's Horizon 2020 research and innovation programme and EFPIA; Norwegian Research Council (no. 31270).

**Research in context**

**Evidence before this study**

Heavy menstrual bleeding (HMB) presents a significant burden for women, substantially impacting their quality of life. We searched PubMed on December 08, 2025, using the terms “heavy menstrual bleeding” and “incidence” and identified 127 studies. Of these, three studies contained relevant incidence rate (IR) results – most other studies cited these three publications, reported prevalence rather than incidence, or were otherwise not relevant as they did not describe the IR of HMB. The three identified studies were UK and Dutch retrospective database analyses that reported IR of HMB ranging from 7.9 to 14.2 per 1000 person-years in a general population of women or adolescents. Two of the three studies additionally characterised common co-occurring conditions, but they were likely insufficiently large to detect rarer co-occurring conditions. While other studies investigating HMB prevalence also described the prevalence of co-occurring conditions, variation in study design and conditions assessed restricted the ability to reliably draw conclusions.

**Added value of this study**

Given that only three studies specifically examined IR of HMB, and these studies varied substantially in design and population characteristics, the existing evidence provides a limited and heterogeneous picture of HMB epidemiology. Our study aimed to provide a clearer and more generalisable real-world understanding of how HMB is identified in real-world data and how identified patients are characterised in clinical settings. We included almost 4 million women with HMB across nine data sources from seven countries, making this, to our knowledge, the largest multinational real-world study to date of women diagnosed with HMB. Using these multinational data, we estimated IR of HMB and prevalence of co-occurring conditions.

**Implications of all the available evidence**

We found that the IR of HMB and the prevalence of co-occurring conditions varied across different healthcare settings covered by the respective data sources. This underscores the importance of considering key data source characteristics when evaluating HMB. In particular, broad coverage across healthcare settings is needed to provide more complete clinical information, along with high-quality, detailed diagnostic coding to enhance the accuracy of the findings. Additionally, we found that the prevalence of certain co-occurring conditions, such as pain, anaemia, and dysmenorrhoea, decreased post-index (ie, after first diagnosis of HMB); these changes in prevalence likely reflect changes in clinical management and may indicate that treatment for HMB could potentially alleviate these conditions. Increased knowledge of HMB and its related conditions, including that derived from our study, may support greater screening for HMB, promote earlier diagnoses, and ultimately improve patient outcomes and quality of life.

## Introduction

Heavy menstrual bleeding (HMB) is defined as excessive menstrual blood loss that interferes with a woman's physical, emotional, social, and material quality of life.^1^, ^2^ These widespread impacts have been reported in studies of varying sizes, countries, and methodologies.^2^, ^3^, ^4^, ^5^, ^6^ HMB and its consequences are often under-recognised and associated with a range of social stigmas, taboos, and negative attitudes that may impede help-seeking, diagnosis, and treatment.^7^, ^8^, ^9^, ^10^ Furthermore, clinicians face challenges in diagnosing HMB, including a reliance on subjective patient recall, variation in what is considered “normal” bleeding, difficulties in accurately gauging the quantity of blood loss, inconsistent nomenclature, and disparity within and between countries in guidelines and services for diagnosis and management.^1^, ^3^, ^11^, ^12^, ^13^, ^14^, ^15^

Few real-world population-based studies have comprehensively characterised women with HMB. Two Dutch database studies, one comprising 881 women consulting their general practitioner for HMB and one comprising 1879 adolescents diagnosed with HMB, reported mean incidence rates (IRs) of 9.3 and 7.9 per 1000 person-years, respectively.^16^, ^17^ The adolescent study also evaluated the prevalence of certain co-occurring conditions, reporting rates of 4.0% for anaemia, 3.5% for iron deficiency anaemia, and 0.0% for endometriosis.^17^ In a further UK database study of 1335 women with von Willebrand disease and 12,463 women without, the IR of HMB was 40.2 per 1000 patient-years and 14.2 per 1000 patient-years, respectively.^18^ The prevalence of co-occurring conditions was somewhat similar for women diagnosed with HMB with vs without concurrent von Willebrand disease: 1.8% vs 2.7% for uterine fibroids, 2.3% vs 3.1% for endometriosis, and 3.4% vs 3.5% for pelvic infections, respectively. The limited sample size of these studies likely prevented detection of rarer co-occurring conditions and contributed to statistical uncertainty around estimates. In addition, the variation in study designs and population characteristics restricted the ability to reliably draw conclusions on the characterisation of HMB.

A clearer real-world understanding of how HMB is characterised in clinical settings is needed to identify potential unmet needs and improve diagnosis and management of both HMB and conditions that may co-occur. Our study, conducted within the European Health Data and Evidence Network (EHDEN),^19^ aimed to address this research gap by assessing the incidence of HMB and prevalence of co-occurring conditions using multinational real-world data.

## Methods

### Study design

This observational cohort study aimed to generate real-world evidence to identify and characterise women diagnosed with HMB using a federated data network approach, on nine real-world data sources from seven countries: Germany, Hungary, the Netherlands, Norway, Spain, the UK, and the USA. These data sources covered differing healthcare settings: primary care only, hospital care only, primary and specialist non-hospital care, all healthcare settings (Table 1). Healthcare setting differences were taken into account in the interpretation of the results.

**Table 1:** Summary of data source characteristics of relevance to studying heavy menstrual bleeding.

CPRD = Clinical Practice Research Datalink. IPCI = Integrated Primary Care Information. IMASIS = Institut Municipal d’Assistència Sanitària Information System. NLHR@UiO = Norwegian Linked Health Registry at University of Oslo. SUCD = Semmelweis University Clinical Data.

The study period was from Jan 1, 2000 (or the earliest available date thereafter) to the end of data availability; end of data availability ranged from 2021 to 2024 across data sources (Table 1). Women aged 11–55 years were included to capture the full reproductive lifespan, encompassing the earliest onset of menstruation through the menopausal transition. For inclusion in the HMB cohort, women must have had continuous observation of 365 days prior to index date (Supplementary Figure S1, Appendix p 6). Index date was defined as the first ever recorded diagnosis of HMB in respective databases. The medical diagnostic codes used to define cases of HMB can be found in Supplementary Table S1 (Appendix p 4). Women were excluded if they had occurrence of any of the following starting on or any time prior to the index date: hysterectomy or bilateral oophorectomy, menopause (induced or natural), or uterine or ovarian cancer. Women were also excluded if they had postcoital bleeding on or 365 days prior to index date, or pregnancy on or 180 days prior to index date. After the index date, women were followed until the earliest occurrence of any of the following: reaching age 55 years, menopause (induced or natural), hysterectomy or bilateral oophorectomy, uterine or ovarian cancer, pregnancy, all-cause mortality, end of data availability, or end of continuous observation in the data source.

### Data sources

Three data sources covered primary care settings only; these were UK Clinical Practice Research Datalink (CPRD) Aurum and CPRD GOLD, as well as Dutch Integrated Primary Care Information (IPCI) (Table 1). IQVIA Disease Analyzer® Germany covered data from primary and non-hospital specialist care. Spanish Hospital del Mar Institut Municipal d'Assistència Sanitària Information System (IMASIS) and Hungarian Semmelweis University Clinical Data (SUCD) covered hospital data. The remaining three data sources – two US claims MarketScan and Optum's de-identified Clinformatics® Data Mart Database (Clinformatics®) and the Norwegian Linked Health Registry at University of Oslo (NLHR@UiO) – covered data from all three healthcare settings (primary care, specialist non-hospital, and hospital).

Data sources were either part of the EHDEN network or available via partner organisations that participated in the EHDEN project. The Observational Medical Outcomes Partnerships common data model was used to standardise medical coding systems and data structure.^20^ This facilitated systematic evidence generation across diverse populations from different sources using a single set of parameterised analytical code while preserving patient privacy.^21^, ^22^

### Ethics

UK data from the CPRD were obtained under licence from the UK Medicines and Healthcare products Regulatory Agency. These data were provided by patients and collected by the NHS as part of their healthcare and support. The use of CPRD data for this study was approved via the Research Data Governance Process of the UK Medicines and Healthcare products Regulatory Agency (protocol 23_003131). Ethical approval for the Norwegian data in this study was obtained from The Regional Committee for Research Ethics (approval number: 155294) and the Data Protection Officer at the University of Oslo (approval number: 523275). IPCI reviewed and approved this study protocol. The remaining data sources needed no approval for use of pseudo-anonymised secondary data. Patient informed consent was not applicable due to the retrospective observational design of the study using de-identified data.

### Demographic covariates and co-occurring conditions

Demographic covariates included age at and year of HMB diagnosis, and race/ethnicity (where available). Conditions of interest (ie, those that often co-occur with HMB) were identified based on guidance from experts and from the literature^23^: adenomyosis, anaemia, coagulopathy, disorder of the ovary, dysmenorrhoea, endometrial hyperplasia, endometriosis, endometriosis of the uterus (presumed adenomyosis), iron deficiency anaemia, obesity, ovulatory dysfunction, pain (defined as general [ie, any pain] and narrow [ie, gynaecological pain that might be correlated with HMB]), pelvic inflammatory disease, polycystic ovary syndrome, sexually transmitted disease, type 2 diabetes, uterine endometrial polyps, and uterine leiomyoma. More information on how covariates were defined and captured can be found in the Supplementary Methods (Appendix p 2). ‘Endometriosis of the uterus’ was included as a potential alternative term for ‘adenomyosis’, as the latter is a relatively recent diagnosis term that may previously have been coded as the former.^24^

We categorised certain co-occurring conditions as “diagnosed conditions likely to underlie HMB” guided by the PALM-COEIN classification system for abnormal uterine bleeding.^23^ These included adenomyosis, endometriosis of the uterus, endometrial hyperplasia, coagulopathy, ovulatory dysfunction, uterine endometrial polyps, and uterine leiomyoma.

### Statistical analysis

We report descriptive analysis of patient demographic and prevalence (as a percentage) of co-occurring conditions among the women diagnosed with HMB. Co-occurring conditions were analysed up to 365 days pre-index date and up to 365 days post-index to detect any changes in their prevalence before and after HMB diagnosis. Additional descriptive comparisons of pre- and post-index estimates are also reported. IRs of HMB with 95% confidence intervals (CIs) in 1000 person-years were determined overall and per calendar year to show any variation over time (calculation details can be found in the Supplementary Methods, Appendix p 3). All analyses were reported for the overall cohort (11–55 years) and were additionally stratified into three age groups (11–29 years, 30–44 years, and 45–55 years). These age ranges were selected due to probable differences in sexual activity and childbearing/family planning needs, particularly when considering treatments for HMB (as will be presented in a subsequent manuscript); for example, we hypothesised that younger women would receive fewer invasive or surgical procedures for HMB than older women, but would likely be more frequently prescribed hormonal contraceptives. We also thought it probable that younger women (<30 years, likely nulliparous) and older women (≥45 years, possibly perimenopausal) would report differences in prevalences of co-occurring conditions. Each data source was analysed and reported separately.

Observational Health Data Sciences and Informatics (OHDSI) tools, Atlas and Athena, were used to develop and test the medical concept sets and the target cohorts.^25^ Data Analysis and Real World Interrogation Network European Union (DARWIN EU) IncidencePrevalence R package v1.2.1 was used for IR analysis.^26^ The following analytical R packages of OHDSI Health Analytics Data-to-Evidence Suite (HADES) were used: CohortDiagnostics, CohortGenerator, CirceR, FeatureExtraction, DatabaseConnector, SqlRender.

### Role of the funding source

This project received funding from the Innovative Medicines Initiative (IMI) 2 Joint Undertaking under grant agreement no. 806968. The Joint Undertaking receives support from the European Union's Horizon 2020 research and innovation programme and EFPIA. Contributions from private consortium partners, including staff time, were provided as in-kind contributions to the IMI EHDEN project. Acquisition of the Norwegian data was funded through Professor Nordeng's grant from the Research Council of Norway, project no. 31270. Bayer CC AG separately funded medical writing assistance for the manuscript. Study design, planning, data analysis, interpretation of the results, and manuscript preparation were undertaken by the study authors, with contributions from staff at participating EHDEN HMB consortium and data partners, as detailed in the Author Contributions statement.

## Results

### Demographic characteristics

Overall, 3,797,918 women with HMB across the nine data sources were included in this study. Patient attrition is shown in Supplementary Figure S2 (Appendix p 7). MarketScan had the largest cohort and SUCD had the smallest (n = 2,000,878 and n = 436, respectively). Median observation time pre- and post-index also varied widely between data source (Table 2). IMASIS had the longest pre-index time and Clinformatics® had the shortest (11.9 and 2.5 years, respectively). NLHR@UiO had the longest post-index time and IQVIA Disease Analyzer® Germany had the shortest (6.8 and 1.0 years, respectively). The median age at first HMB diagnosis was relatively similar across the data sources, ranging from 38 to 42 years old (Table 2).

**Table 2:** Participant characteristics at first diagnosis of heavy menstrual bleeding, aged 11–55 years, in the US, UK, and Europe.

CPRD = Clinical Practice Research Datalink. IPCI = Integrated Primary Care Information. IMASIS = Institut Municipal d’Assistència Sanitària Information System. IQR = interquartile range. NLHR@UiO = Norwegian Linked Health Registry at University of Oslo. SUCD = Semmelweis University Clinical Data.

^∗^Only Clinformatics® had available race and ethnicity data: they reported 63.8% White, 12.0% Black or African American, 4.5% Asian, and 19.7% unknown. In total, 12.8% were Hispanic or Latina, 80.4% were not Hispanic or Latina, and 6.8% were unknown.

### Incidence rates of HMB

The overall IR of HMB per 1000 person-years (95% CI) ranged from 0.7 (0.6, 0.7) in SUCD (hospital only) to 25.9 (25.8, 25.9) in Clinformatics® (all healthcare settings) (Fig. 1; Supplementary Table S2, Appendix p 5). Data sources that included all healthcare settings reported the highest IRs, followed by IQVIA Disease Analyzer® Germany – a data source that included primary and specialist non-hospital care. In contrast, primary care-only data sources reported lower IRs, and hospital-only data sources reported the lowest IR (Fig. 1).

**Fig. 1:**
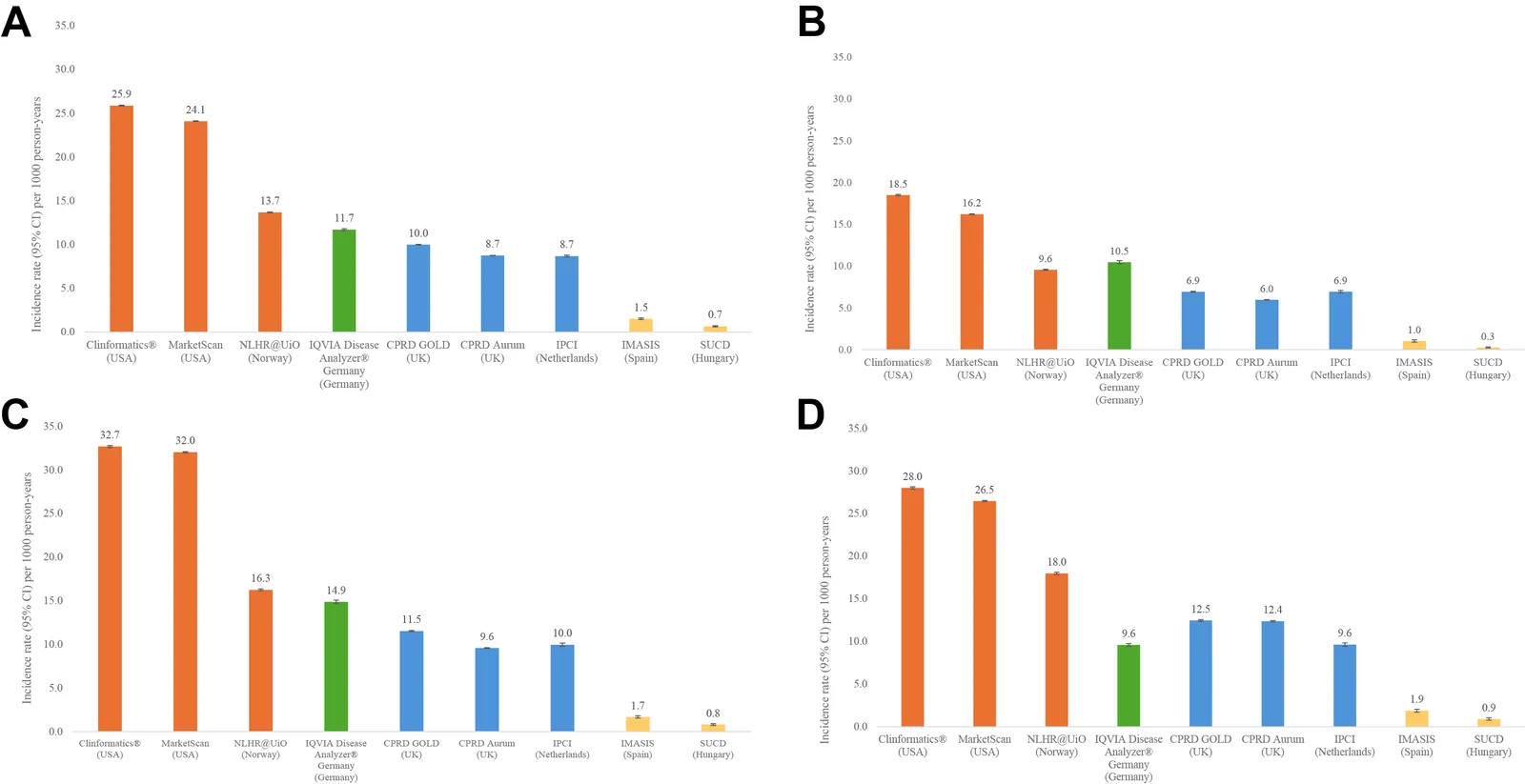
**Overall incidence rates, per 1000 person-years, of heavy menstrual bleeding for each data source across the entire observation period for A) all ages, B) 11–29 years, C) 30–44 years, and D) 45–55 years**. CI = Confidence Interval. CPRD = Clinical Practice Research Datalink. IPCI = Integrated Primary Care Information. IMASIS = Institut Municipal d’Assistència Sanitària Information System. NLHR@UiO = Norwegian Linked Health Registry at University of Oslo. SUCD = Semmelweis University Clinical Data.

Observing annual IR trends, the two US data sources covering all healthcare settings showed a marked increase in IR between 2014 and 2016 (Supplementary Figure S3, Appendix p 8). NLHR@UiO (also all healthcare settings) exhibited a slight increase in IR toward the end of the study period. In contrast, annual IRs in primary care-only data sources remained relatively stable, with the exception of a slightly decreasing trend observed in CPRD GOLD. IMASIS, a data source including hospital-only care, reported a marked increase in IR from 2017.

When stratified by age, overall IRs (95% CI) were highest in the 30–44-year age group across most data sources, ranging from 0.8 (0.7, 0.9) in SUCD to 32.7 (32.6, 32.8) per 1000 person-years in Clinformatics® (Fig. 1; Supplementary Table S2, Appendix p 5). IRs were lowest in the youngest age group (11–29 years), ranging from 0.3 (0.2, 0.3) in SUCD to 18.5 (18.4, 18.6) per 1000 person-years in Clinformatics®. The younger age group showed an increasing annual IR trend across all data sources, except for SUCD (where the sample size might be insufficient to establish reliable trends across age groups) and the IQVIA Disease Analyzer® Germany data source (which lacked sufficient data availability to determine trends) (Supplementary Figure S4, Appendix p 9). IRs in the oldest age group (45–55 years) ranged from 0.9 (0.9, 1.1) in SUCD to 28.0 (27.9, 28.1) per 1000 person-years in Clinformatics®. This age group had a slight decrease in annual IR in data sources covering only primary care (Supplementary Figure S4, Appendix p 11).

### Prevalence of co-occurring conditions up to 365 days prior to index date

Of the diagnosed conditions considered most likely to underlie HMB, uterine leiomyoma was most prevalent across all data sources in the 365 days prior to the index date, with markedly lower prevalence observed in primary care-only data sources (Fig. 2). Prevalence ranged from 0.9% in CPRD GOLD to 10.5% in IMASIS. This was followed by endometrial polyps, although prevalence of this was only comparatively high in the two hospital data sources (4.0% for IMASIS and 7.6% for SUCD), and ovulatory dysfunction, which ranged from 0.6–0.7% in the primary care-only sources to 3.9–4.4% in the US data sources. Coagulopathy, endometrial hyperplasia, and endometriosis of the uterus were uncommon across the data sources, peaking at 0.5% in SUCD, 2.5% in SUCD, and 1.3% in IMASIS, respectively. Adenomyosis was rare, observed only in a few cases in Clinformatics® and not reported in the other data sources.

**Fig. 2:**
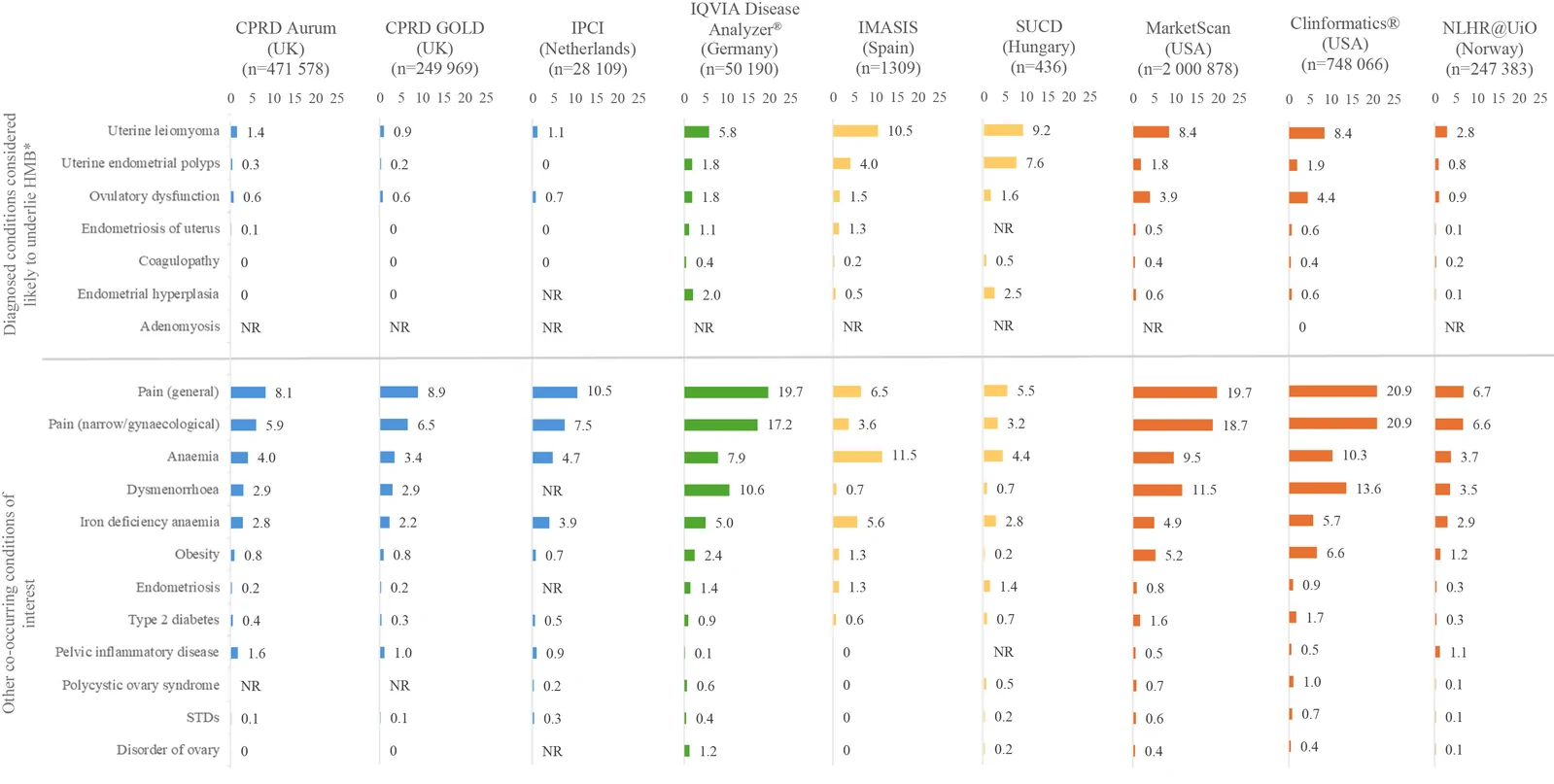
**Prevalence (%) of co-occurring conditions, in women with heavy menstrual bleeding aged 11–55 years, 365 days before index date**. ∗Categorisation of diagnosed conditions considered likely to underlie HMB was guided by the PALM-COEIN classification system for abnormal uterine bleeding.^23^ CPRD = Clinical Practice Research Datalink. HMB = heavy menstrual bleeding. IPCI = Integrated Primary Care Information. IMASIS = Institut Municipal d’Assistència Sanitària Information System. NLHR@UiO = Norwegian Linked Health Registry at University of Oslo. NR = not recorded. STD = sexually transmitted disease. SUCD = Semmelweis University Clinical Data.

Among the remaining co-occurring conditions, pain (using both general and narrow/gynaecological definitions) was most prevalent across all data sources except for IMASIS (hospital only), where it ranked second after anaemia (Fig. 2). Prevalence of pain was highest in Clinformatics® (20.9% for both general and narrow/gynaecological pain) and lowest in SUCD (5.5% for general and 3.2% for narrow/gynaecological pain). The prevalence of anaemia, including iron deficiency anaemia, also ranked relatively high, regardless of the healthcare setting, ranging from 3.4% (CPRD GOLD) to 11.5% (IMASIS) for anaemia, and from 2.2% (CPRD GOLD) to 5.7% (Clinformatics®) for iron deficiency anaemia. In addition, dysmenorrhoea was most prevalent in the US and German data sources (10.6–13.6%) but rare in the two hospital data sources (both 0.7%).

When stratified by age, the prevalence of conditions considered most likely to underlie HMB was substantially lower in the youngest age group (11–29 years), regardless of the healthcare setting (Supplementary Figure S5, Appendix pp 12–14). Uterine leiomyoma remained the most frequently diagnosed condition considered likely to underlie HMB in the 30–44-year (ranging from 0.9% in CPRD GOLD to 11.3% in IMASIS) and the 45–55-year age groups (ranging from 1.8% in CPRD GOLD to 15.7% in Clinformatics®). Among the remaining conditions, pain (general and narrow/gynaecological) was the most prevalent across all age groups and was slightly more common among the youngest women (11–29 years); general pain peaked at 24.8% for women aged 11–29 years, 19.6% for 30–44 years, and 20.9% for 45–55 years (all in Clinformatics®). The prevalence of dysmenorrhoea decreased markedly with age, peaking at 20.8% in 11–29 years, 12.5% in 30–44 years, and 7.7% in 45–55 years (all in Clinformatics®). Anaemia, on the other hand, exhibited the opposite pattern, increasing in prevalence among older women; it peaked at 10.5% in 11–29 years (IMASIS), 12.2% in 30–44 years (IMASIS), and 13.3% in 45–55 years (Clinformatics®).

### Prevalence of co-occurring conditions up to 365 days after index date

Of the diagnosed conditions considered most likely to underlie HMB, uterine leiomyoma remained among the most prevalent in the 365 days after the index date, with prevalence ranging from 1.1% in IPCI to 9.1% in MarketScan (Fig. 3). Compared with the pre-index period, the post-index prevalence more than halved in hospital-only data sources and the data source covering primary plus specialist non-hospital care. However, in primary care-only data sources and data sources covering all healthcare settings, prevalence increased or did not change. Post-index, the prevalence of endometrial polyps increased across all data sources except for IQVIA Disease Analyzer® Germany and the two hospital sources; this was the second most common diagnosed condition considered likely to underlie HMB in the US data sources (7.6–8.2%), increasing more than fourfold compared with pre-index prevalences. The prevalence of ovulatory dysfunction remained largely unchanged, except for a slight decrease in the two US data sources; its prevalence ranged from 0.6% in IMASIS and IPCI to 3.0% in Clinformatics®. The prevalence of endometriosis of the uterus increased post-index in the US data sources, with prevalences of 3.7% in both. However, this increase was not observed in the other data sources. Coagulopathy, endometrial hyperplasia, and adenomyosis remained uncommon, at <1.5% across all data sources.

**Fig. 3:**
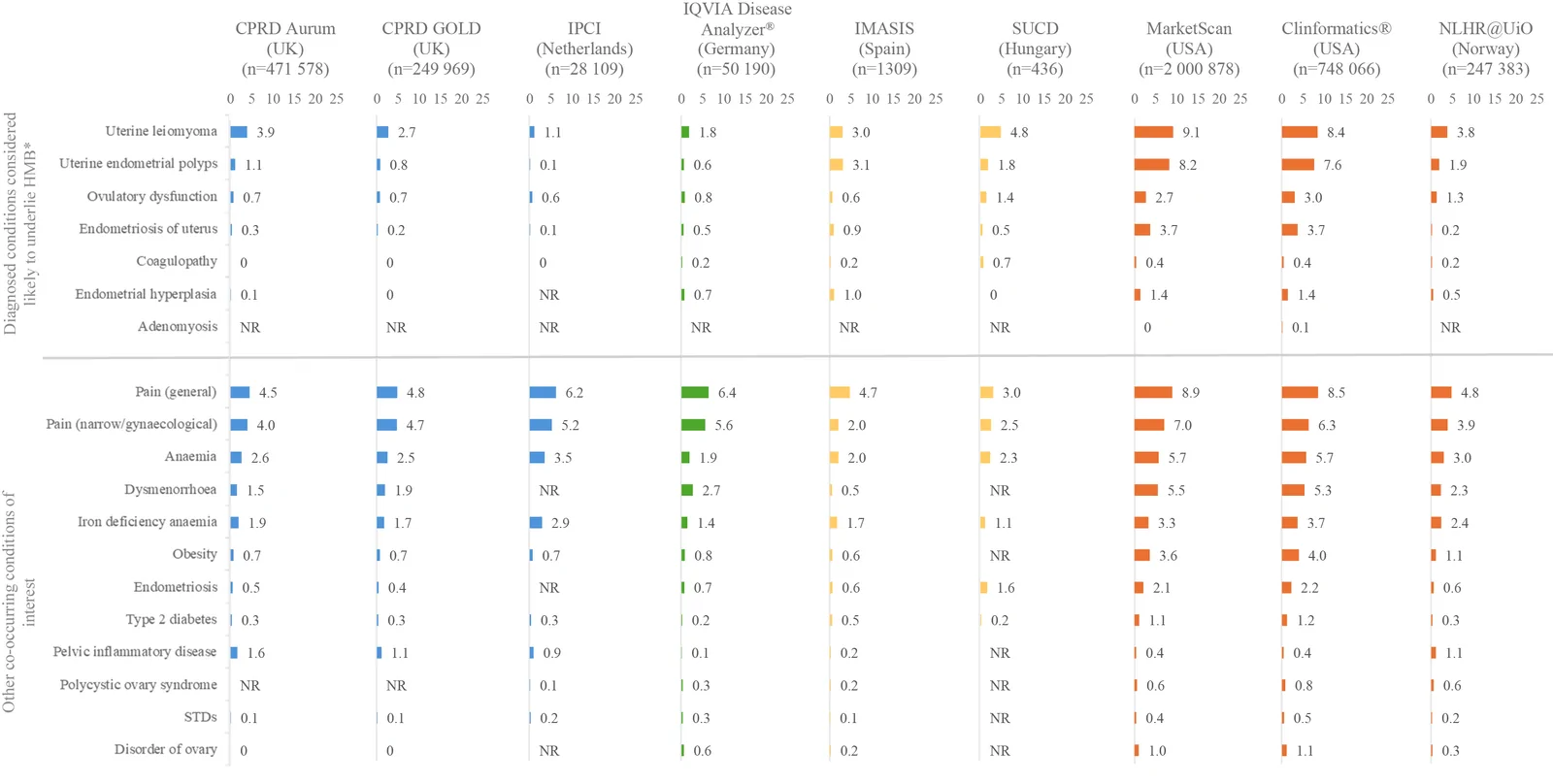
**Prevalence (%) of co-occurring conditions, in women with heavy menstrual bleeding aged 11–55 years, 365 days after index date**. ∗Categorisation of diagnosed conditions considered likely to underlie HMB was guided by the PALM-COEIN classification system for abnormal uterine bleeding.^23^ CPRD = Clinical Practice Research Datalink. HMB = heavy menstrual bleeding. IPCI = Integrated Primary Care Information. IMASIS = Institut Municipal d’Assistència Sanitària Information System. NLHR@UiO = Norwegian Linked Health Registry at University of Oslo. NR = not recorded. STD = sexually transmitted disease. SUCD = Semmelweis University Clinical Data.

Among the remaining co-occurring conditions, pain (general and narrow/gynaecological) continued to be most common across all data sources (Fig. 3), with prevalences ranging from 3.0 in SUCD to 8.9% in MarketScan for general pain, and from 2.0 in IMASIS to 7.0% in MarketScan for narrow/gynaecological pain. However, the prevalence of pain was substantially lower post-index event across all data sources, most notably in the US data sources and IQVIA Disease Analyzer® Germany. Additionally, across all data sources, there was a substantial decrease of approximately 50% in the prevalence of anaemia and iron deficiency anaemia compared with pre-index. The prevalence of dysmenorrhoea remained highest in the US and German data sources (2.7–5.5%); however, this condition also underwent a substantial decrease compared with pre-index across all data sources. Conversely, the prevalence of endometriosis increased in the US data sources when compared with pre-index (post-index prevalence of 2.1–2.2%). A less pronounced increase in the prevalence of endometriosis was observed in all other data sources, except IQVIA Disease Analyzer® Germany and IMASIS where prevalence decreased.

When stratifying by age, the prevalence of conditions considered most likely to underlie HMB remained relatively uncommon post-index in the youngest age group (11–29 years), whereas pain remained common across all age groups post-index date (Supplementary Figure S6, Appendix pp 15–17). The previously observed pre-index pattern of a reduction in pain and dysmenorrhoea prevalence with increasing age could no longer be observed post-index. However, the observed pattern of higher anaemia prevalence with increasing age was still evident. General pain peaked at 7.5% for women aged 11–29 years (MarketScan), 9.3% for 30–44 years (MarketScan), and 9.5% for 45–55 years (MarketScan and Clinformatics®). Dysmenorrhoea peaked at 4.9% (MarketScan), 6.3% (MarketScan), and 4.9% (Clinformatics®), respectively. Anaemia peaked at 3.4% (MarketScan and Clinformatics®), 5.9% (MarketScan and Clinformatics®), and 7.7% (Clinformatics®), respectively.

## Discussion

To our knowledge, the EHDEN HMB study was the largest multinational real-world study to date of women diagnosed with HMB. It included almost 4 million women with HMB from nine data sources across seven countries. We reported the overall and annual IR of HMB and described patient characteristics and the prevalence of co-occurring conditions.

This study emphasised the importance of considering data source characteristics, including sample size and coverage across healthcare settings, in database studies as these factors influenced the observed results. Data sources that covered all healthcare settings reported the highest IRs of HMB, while hospital-only data sources reported the lowest IRs. In addition to the variability in IR, differences in patterns of co-occurring conditions were also evident across data sources. Pain, anaemia, and dysmenorrhoea were among the most common co-occurring conditions both pre- and post-index regardless of the data source. In contrast, more complex disorders exhibited greater variation between data sources, with low prevalences especially in primary care-only data sources, likely reflecting that these conditions tend to be managed in specialist care settings. For example, uterine leiomyoma was the most prevalent diagnosed condition considered likely to underlie HMB, but prevalence differed markedly between the data sources.

In this study, several co-occurring conditions considered likely to underlie HMB were infrequently recorded across all data sources. This contrasts with substantially higher prevalences reported in the literature. For example, systematic reviews reported prevalences of endometrial hyperplasia in pre-menopausal women with abnormal uterine bleeding ranging from 3.4% to 25.6%.^27^ The lower prevalences observed in our study should be interpreted with caution, as many published estimates originate from specialist clinics evaluating highly selected populations of women with bleeding symptoms, who may be more likely to undergo diagnostic work-up for underlying conditions than women identified through routine healthcare data bases. Similarly, adenomyosis was rarely captured in our data despite retrospective hospital-based studies in China from 2019 to 2021 reporting prevalences of approximately 10%.^28^ Contributing factors to these discrepancies may include incomplete or inconsistent diagnostic coding, the relatively recent introduction of specific codes for adenomyosis (introduced in 2022), and diagnostic pathways that rely on imaging or surgical confirmation. Such investigations are not uniformly available across healthcare settings or may be inconsistently recorded in routine data. These findings highlight the importance of high-quality, detailed diagnostic coding to accurately characterise HMB and its underlying conditions in real-world data.

In our study, five of the nine data sources reported an IR of HMB of 8–14%, which is similar to the previously described rates found in the Dutch and UK retrospective database analyses.^16^, ^17^, ^18^ The two US data sources had higher IR, while the two hospital-only sources had lower IR. Post-index prevalences of co-occurring conditions were also consistent with those prior findings, although US data sources more frequently reported conditions such as uterine leiomyoma.^17^, ^18^ Additionally, IR were highest in women aged 30–44 years, consistent with the UK study reporting peak incidence in women aged 30–49 years.^18^ One possible explanation for this pattern is that hormonal contraceptive use during earlier reproductive years may reduce or suppress menstrual bleeding, potentially delaying recognition and diagnosis of underlying HMB until later reproductive years. Another potential explanation is that many women experience HMB for extended periods before seeking medical help or receiving a formal diagnosis; some may choose to self-manage their symptoms and thus would remain uncaptured. This interpretation is supported by a prior survey-based study among 1000 women with HMB, in which 40% had never consulted a clinician about their HMB symptoms. The average delay between symptom onset and seeking medical help was approximately 3 years, and around two-thirds did not receive a formal diagnosis even after discussing their HMB symptoms with their healthcare provider.^3^

The prevalence of most co-occurring conditions changed before and after the index date, which may reflect changes in treatment plans following an HMB diagnosis. The substantial post-index decrease in prevalence of conditions such as pain, anaemia, and dysmenorrhoea suggests that these conditions could be associated with HMB and potentially alleviated by treatment for HMB. On the other hand, the post-index increases in some conditions in certain data sources (such as uterine leiomyoma in primary care-only data sources, and endometrial hyperplasia and endometriosis in the US data sources) may indicate that the physician took steps to further investigate the underlying cause of the HMB.

Leveraging the EHDEN network and using a federated network approach allowed us to generate standardised evidence from diverse countries and healthcare settings. However, as mentioned, caution is needed when interpreting differences, as results may reflect characteristics inherent to the data itself, rather than genuine clinical heterogeneity between women with HMB.

Beyond cross-data source variability, several limitations relate to the underlying data. Some aspects of clinical heterogeneity, such as race and ethnicity, could not be analysed as these data were largely unavailable. Moreover, the index event, defined as the first recorded code of HMB in a woman's clinical record, reflects what is reported in databases but may not represent the first occurrence of HMB or the first clinical consultation. Supporting this, conditions such as anaemia were frequently recorded prior to HMB diagnosis. Additionally, underdiagnosis and incomplete capture in routine data may lead to underestimation of the true burden of HMB. Furthermore, age-based identification, rather than confirmed reproductive status, may dilute the population at risk, as some women in the denominator may not have been biologically at risk throughout the observation period, e.g. pre-menarcheal or post-menopausal women. This may also impact estimation of IRs. The CIs of the IRs were calculated using exact methods. Potential overdispersion was not formally assessed and, if present, uncertainty around the IR estimates may therefore be underestimated.^29^ Finally, some of the post-index decreases in co-occurring conditions such as pain and anaemia may reflect reduced use of symptom codes once an underlying diagnosis (e.g., uterine leiomyoma or adenomyosis) has been established.

In this study, we employed the PALM-COIEN system to assess co-occurring diagnosed conditions likely to underlie HMB.^23^ However, this classification system was developed for abnormal uterine bleeding more broadly, rather than HMB specifically. As such, some categories are less relevant for HMB; for example, iatrogenic bleeding associated with hormonal medication more commonly presents as unscheduled bleeding or spotting, rather than as chronic HMB.^30^ Consequently, the contribution of iatrogenic causes to HMB was not evaluated in the present study.

In conclusion, this large-scale, multinational, real-world study characterised the incidence of HMB and prevalence of its co-occurring conditions. The wide variation in observed IRs and prevalence estimates across countries, healthcare settings, and data sources highlights how differences in care delivery and clinical records could influence HMB detection and characterisation. Pain, anaemia, and dysmenorrhoea were the most common co-occurring conditions overall, while uterine leiomyoma was the common diagnosed condition considered to underlie HMB. The low prevalences reported for many of the other co-occurring conditions may indicate suboptimal data capture or diagnosis. Changes in prevalence of co-occurring conditions before and after the index date likely reflect both clinical management and diagnostic investigation following HMB identification. We intend to explore the treatments and procedures used by women before and after HMB diagnosis in a subsequent manuscript.

Overall, our results emphasise the importance of using data sources with broad healthcare coverage and high-quality, detailed coding to more accurately characterise HMB in real-world settings. Increased awareness and improved understanding of HMB and its related conditions, including insights derived from our study, may support more systematic screening, promote earlier diagnoses, and ultimately improve patient outcomes and quality of life.

## Contributors

All authors read and approved the final version of the manuscript. Data verification was performed by: Hedvig Nordeng, Nhung TH Trinh, Saeed Hayati (Norway); Miguel-Angel Mayer, Angela Leis, Juan Manuel Ramírez Anguita (Spain); Katia Verhamme (Netherlands); Loretta Zsuzsa Kiss, Zsolt Bagyura (Hungary); Ronald Herrera (Germany); Gianmario Candore, Carina Dinkel-Keuthage, Katrin Manlik, George Argyriou (UK & USA).

Marta Pineda-Moncusi was involved in the study conduct, data analysis and interpretation, and manuscript writing of this study.

Zsolt Bagyura was involved in data analysis and interpretation, and manuscript writing of this study.

Carina Dinkel-Keuthage was involved in the study conduct, data analysis and interpretation, and manuscript writing of this study.

Kristina Gemzell-Danielsson was involved in data analysis and interpretation, and manuscript writing of this study.

Saeed Hayati performed the data analyses of the Norwegian data in this study, including the mapping of data onto the OMOP CDM. He was involved in data interpretation, and manuscript writing of this study.

Oskari Heikinheimo was involved in data analysis and interpretation, and manuscript writing of this study.

Ronald Herrera was involved in data analysis and interpretation, and manuscript writing of this study.

Bruno Imthurn was involved in the study design, planning, data analysis and interpretation, and manuscript writing of this study.

Sara Khalid was involved in the data analysis and interpretation, and manuscript writing of this study.

Loretta Zsuzsa Kiss was involved in the data analysis and interpretation, and manuscript writing of this study.

Angela Leis was involved in the data analysis and interpretation, and manuscript writing of this study.

Katrin Manlik was involved in the study conduct, data analysis and interpretation, and manuscript writing of this study.

Miguel-Angel Mayer was involved in the data analysis and interpretation, and manuscript writing of this study.

Carsten Möller was involved in the study design, planning, and conduct, data analysis and interpretation, and manuscript writing of this study.

Juan Manuel Ramírez Anguita was involved in the data analysis and interpretation, and manuscript writing of this study.

Hedvig Nordeng was involved in the study design, data analysis and interpretation, and manuscript writing of this study. She had overall responsibility for the Norwegian data in this study, including obtaining all required approvals and overseeing the mapping to the OMOP CDM.

Siir Su Saydam was involved in the data analysis and interpretation, and manuscript writing of this study.

Mariana Shiffer Acar was involved in study conduct, data analysis and interpretation, and manuscript writing of this study.

Nhung TH Trinh was involved in the study design, data analysis and interpretation, and manuscript writing of this study.

George Argyriou was involved in the study design, planning, and conduct, data analysis and interpretation, and manuscript writing of this study.

Katia Verhamme was involved in the data analysis and interpretation, and manuscript writing of this study.

Gianmario Candore was involved in the study design, planning, and conduct, data analysis and interpretation, and manuscript writing of this study.

## Data sharing statement

All relevant aggregated data supporting the findings of this study are presented within the article and its Supporting Information files. Individual-level registry data cannot be shared by the authors due to legal and ethical restrictions governing access to health registry data in each country. Researchers may apply for permission to use the data through the relevant registry holders in each country, subject to approval from relevant registry holders and appropriate ethical and data protection authorities.

## Declaration of interests

Kristina Gemzell-Danielsson has received honoraria for ad hoc lectures and participation in advisory meetings for Bayer AG, Organon, Exeltis, Gedeon Richter, MedinCell, Exelgyn, HRA-Pharma and Natural cycles.

Oskari Heikinheimo has received travel support from Gedeon Richter and consulting fees from Orion Pharma and Nordic Pharma. Oskari Heikinheimo has also served on advisory boards and helped design educational events for Bayer AG and Gedeon Richter.

Ronald Herrera was an employee of Boehringer-Ingelheim and Bayer AG during the conception, conduct and analysis of this study.

Bruno Imthurn receives funding from Bayer as member of the LCS12 Safety Monitoring and Advisory Council.

Sara Khalid receives funding from Wellcome Trust, Gates Foundation, and UKRI outside of this work.

Marta Pineda-Moncusi, Zsolt Bagyura, Saeed Hayati, Loretta Zsuzsa Kiss, Angela Leis, Miguel-Angel Mayer, Juan Manuel Ramírez Anguita, Hedvig Nordeng, Nhung TH Trinh, George Argyriou, and Katia Verhamme have no conflicts of interest to declare.

Siir Su Saydam and Gianmario Candore were employees of Bayer during the conduct and analysis of this study.

Carina Dinkel-Keuthage, Katrin Manlik, Carsten Möller, and Mariana Schiffer Acar are all current employees of Bayer.

Bayer funded the medical writing assistance of this manuscript. Bayer has several products indicated for treatment of HMB.
