# Supplementary material for: Incidence of heavy menstrual bleeding and prevalence of co-occurring conditions from seven countries across Europe and North America: an EHDEN real-world data study
Source: eClinicalMedicine. 2026 Nov 3;101:104297. doi: 10.1016/j.eclinm.2026.104297 (PMC13756298; doi:10.1016/j.eclinm.2026.104297)
Supplement: Supplementary file 1 [file mmc1.docx]

**Supplementary Information**

Incidence of heavy menstrual bleeding and co-occurring conditions from seven countries across Europe and North America: an EHDEN real-world data study

Marta Pineda-Moncusi, Zsolt Bagyura, Carina Dinkel-Keuthage, Kristina Gemzell-Danielsson, Saeed Hayati, Oskari Heikinheimo, Ronald Ferney Herrera Clavijo, Bruno Imthurn, Sara Khalid, Loretta Zsuzsa Kiss, Angela Leis, Katrin Manlik, Miguel-Angel Mayer, Carsten Möller, Juan Manuel Ramírez Anguita, Nhung TH Trinh, Siir Su Saydam, Mariana Schiffer Acar, Hedvig Nordeng, George Argyriou, Katia Verhamme, Gianmario Candore

| **Contents** | **Page** |
| --- | --- |
| Supplementary methods | 2 |
| Statement of ethics | 2 |
| Definition of disorder of the ovary | 2 |
| Conditions classified as STDs | 2 |
| Definition of incidence rate (IR) | 3 |
| Supplementary tables | 4 |
| *Supplementary Table 1:* Medical coding of heavy menstrual bleeding | 4 |
| *Supplementary Table 2:* Overall incidence rates, per 1000 person-years, of heavy menstrual bleeding per data source across all years of observation in the US, UK, and Europe | 5 |
| Supplementary figures | 6 |
| *Supplementary Figure 1:* Study design | 6 |
| *Supplementary Figure 2:* Patient attrition (number of patients per selection stage) for each data source | 7 |
| *Supplementary Figure 3:* Annual incidence rates, per 1000 person-years, of heavy menstrual bleeding for each data source | 8 |
| *Supplementary Figure 4:* Annual incidence rates, per 1000 person-years, of heavy menstrual bleeding for each data source among women A) aged 11 to 29 years, B) aged 30 to 44 years, and C) aged 45 to 55 years | 9 |
| *Supplementary Figure 5:* Prevalence (%) of co-occurring conditions in women with heavy menstrual bleeding 365 days before index date among women A) aged 11 to 29 years, B) aged 30 to 44 years, and C) aged 45 to 55 years | 12 |
| *Supplementary Figure 6:* Prevalence (%) of co-occurring conditions in women with heavy menstrual bleeding 365 days after index date among women A) aged 11 to 29 years, B) aged 30 to 44 years, and C) aged 45 to 55 years | 15 |

Supplementary Methods

Definition of disorder of the ovary

The definition “disorder of the ovary” encompassed a wide range of conditions and disorders: abscess of ovary; acute oophoritis; acute salpingo-oophoritis; bilateral endometriosis of ovaries; chronic oophoritis; chronic salpingo-oophoritis; combined intrauterine and ovarian pregnancy; corpus luteum deficiency syndrome; cycling ovulatory disorder; disorder of endocrine ovary; disorder of fallopian tube and ovary; disorder of ovary; endocrine menstrual disturbance; endometriosis of ovary; endometriosis of left ovary; endometriosis of right ovary; female hypogonadism syndrome; female hypogonadotropic hypogonadism; female infertility due to diminished ovarian reserve; female infertility due to oligo-ovulation; female infertility due to ovulatory disorder; female infertility of anovulatory origin; gonococcal tubo-ovarian abscess; hyperoestrogenism; hypergonadotropic amenorrhoea; hyperhormonal amenorrhoea; hyperplasia of ovarian stroma; hypersecretion of ovarian androgens; hypersecretion of ovarian progesterone; hypertrophy of ovary; hypothalamic amenorrhoea; infection of ovary; isosexual virilisation; large ovary; left tubo-ovarian abscess; lesion of left ovary; lesion of ovary; lesion of right ovary; luteal phase defect; luteinised unruptured follicle syndrome; massive ovarian oedema; mumps oophoritis; non-inflammatory disorder of ovary; oophoritis; ovarian hyperfunction; ovarian hypersecretion; ovarian hyperthecosis; primary anovulatory infertility; prolapse of ovary; retained corpus luteum; right tubo-ovarian abscess; salpingo-oophoritis following abortive pregnancy; salpingo-oophoritis following ectopic pregnancy; salpingo-oophoritis following molar pregnancy; secondary anovulatory infertility; subacute oophoritis; subacute salpingo-oophoritis; superficial endometriosis of ovary; tuberculous oophoritis; tubo-ovarian abscess; tubo-ovarian inflammatory disease.

Conditions classified as STDs

Medical coding terms classified as STDs included: Abscess gonococcal, abscess of urethral gland due to *Neisseria gonorrhoeae*, acquired syphilis, acute gonococcal, acute gonorrhoea, acute myocarditis – syphilitic, acute secondary syphilitic meningitis, acute syphilitic meningitis, acute syphilitic pericarditis, adult gonococcal conjunctivitis, anetoderma secondary to syphilis, anogenital human papillomavirus infection, anogenital papillomaviral intraepithelial neoplasia, asymptomatic neurosyphilis, asymptomatic secondary neurosyphilis, bilateral chorioretinitis of eyes caused by secondary syphilis, bilateral iritis of eyes caused by *Treponema pallidum*, bilateral retinitis of eyes caused by secondary syphilis, bilateral retinitis of eyes caused by *Treponema pallidum*, bursitis caused by late syphilis, cardiovascular syphilis, chancroid, *Chlamydia trachomatis* infection in pregnancy, chorioretinitis of left eye caused by secondary syphilis, chorioretinitis of right eye caused by secondary syphilis, chronic gonococcal, chronic gonorrhoea, Clutton’s joints, complicated donovanosis, condyloma latum, congenital syphilis, congenital syphilitic, corona veneris, cutaneous gonorrhoea, dementia paralytica juvenilis, disseminated infection caused by *Neisseria gonorrhoeae*, donovanosis – anogenital ulcer, donovanosis – non-genital lesion, early congenital syphilis, early congenital syphilis – latent, early lymphogranuloma venereum, early symptomatic syphilis, endocochlear syphilis, esthiomene, female gonococcal pelvic inflammatory disease, female syphilitic pelvic inflammatory disease, general paresis – neurosyphilis, genital herpes simplex, gingival disease caused by *Neisseria gonorrhoeae*, gingival disease caused by *Treponema pallidum*, gonococcal, gonococcemia, gonorrhoea, granuloma inguinale, gummatous neurosyphilis, hepatitis caused by sexually transmissible virus, hepatitis in late syphilis, hepatitis in secondary syphilis, herpes simplex of female genitalia, herpetic cervicitis, herpetic ulceration of vulva, herpetic vesicle in vagina, herpetic vulvovaginitis, Hutchinson’s teeth, Hutchinson’s triad, infection of anus and rectum caused by *Neisseria gonorrhoeae*, infestation by *Pthirus pubis*, interstitial glossitis, iris papulosa caused by *Treponema pallidum*, iris roseola caused by *Treponema pallidum*, iritis of left eye caused by *Treponema pallidum*, iritis of right eye caused by *Treponema pallidum*, juvenile tabes dorsalis, juvenile taboparesis, keratitis due to syphilis, late congenital cardiovascular syphilis, late congenital neurosyphilis, late congenital syphilis, late congenital syphilitic, late latent syphilis, late lymphogranuloma venereum, late quaternary neurosyphilis, late secondary syphilis, late syphilis, late syphilitic, latent early syphilis, latent syphilis, localised cutaneous gonococcal infection, lues maligna, luetic glossitis, lymphogranuloma venereum, macular syphilide, maternal gonorrhoea, meningovascular syphilis - quaternary stage, Moon’s molar teeth, mucocutaneous early congenital syphilis, nasal syphilis, neonatal gonococcal infection, neonatal gonococcal vulvovaginitis, neurosyphilis, nodular syphilide, non-ulcerative sexually transmitted infection caused by *Chlamydia trachomatis*, ocular late syphilis, ocular syphilis, oral mucous membrane lesion due to late syphilis, oral syphilis, otosyphilis, papular syphilide, penile chancroid, periurethral abscess caused by *Neisseria gonorrhoeae*, primary anal syphilis, primary extragenital syphilis, primary genital syphilis, primary herpes simplex infection of genitalia, primary herpetic vulvovaginitis, primary symptomatic early syphilis, primary syphilis, primary syphilis of breast, primary syphilis of fingers, primary syphilis of lip, primary syphilis of nipple, primary syphilis of tonsils, progressive locomotor ataxia, pustular syphilide, quaternary syphilis, rash of secondary syphilis, recurrent genital herpes simplex, recurrent genital herpes simplex type 1 infection, recurrent genital herpes simplex type 2 infection, recurrent herpes simplex infection of buttock, recurrent herpetic vulvovaginitis, rupture of syphilitic cerebral aneurysm, secondary neurosyphilis, secondary symptomatic early syphilis, secondary syphilis, secondary syphilis relapse, secondary syphilitic adenopathy, secondary syphilitic chorioretinitis, secondary syphilitic meningitis, secondary syphilitic periostitis, serological relapse after treatment of latent early syphilis, sexually transmissible infection caused by herpes simplex virus, sexually transmissible infection caused by human papillomavirus, sexually transmissible infection caused by Molluscum contagiosum virus, sexually transmitted bacterial disease affecting skin, sexually transmitted infectious disease, sexually transmitted infectious disease in mother complicating childbirth, spastic spinal syphilitic paralysis, symptomatic late congenital syphilis, syphilis, syphilitic, tabetic joint, tabetic neurosyphilis, taboparesis, tertiary syphilitic chorioretinitis, trichomonal cystitis, trichomonal vaginitis, trichomonal vulvitis, urethral stricture due to gonococcal infection, urogenital infection by *Trichomonas vaginalis*, uveitis due to secondary syphilis, venereal disease due to *Chlamydia trachomatis*, venereal disease in mother complicating pregnancy, childbirth AND/OR puerperium, venereal disease in pregnancy, vulval chancroid.

Definition of incidence rate (IR)

IRs of HMB in women of reproductive age per year and over the entire study period were calculated. For this, the denominator consisted of women aged 11 to 55 years with a visit occurrence on or after the 1st of January of 2000. Women in the denominator cohort were also required to have a minimum of 365 days of available observation prior to entry and no record of hysterectomy, bilateral oophorectomy, or a menopause (natural or induced) diagnosis in the patient records before study entry date.

Female patients were followed up until the first of the following:

- end of continuous observation in the database
- a visit occurrence at age > 55 years
- menopause (induced or natural)
- hysterectomy or bilateral oophorectomy
- uterine or ovarian cancer
- all-cause mortality

Incident cases were identified as the first recorded occurrence of HMB after study entry. When an incident HMB event occurred, the individual contributed one event to the numerator of the incidence rate and no longer contributed their person-time to the denominator.

Incidence rates were computed as follows:

IR = (Number of new cases in time period) / (Total person time of the at-risk population) × 1000

Overall and annual IRs were calculated, per 1000 person-years, together with 95% exact confidence intervals.^1^ IRs were further stratified by age groups (11 to 29 years, 30 to 44 years, 45 to 55 years). The package IncidencePrevalence was used for these calculations.^2,3^

**References**

1	Clopper CJ, Pearson ES. The use of confidence or fiducial limits illustrated in the case of the binomial. Biometrika 1934; 26: 404–13.

2	IncidencePrevalence R package. Available at: <https://onlinelibrary.wiley.com/doi/10.1002/pds.5717> [Accessed July 2026].

3	Raventós B, Català M, Du M, et al. IncidencePrevalence: An R package to calculate population-level incidence rates and prevalence using the OMOP common data model. Pharmacoepidemiol Drug Saf. 2024; 33: e5717.

Supplementary tables

*Supplementary Table 1:* Medical coding of heavy menstrual bleeding

| **ID** | **Code** | **Name** | **Domain** | **Vocabulary** |
| --- | --- | --- | --- | --- |
| **HMB source codes** | | |  |  |
| 35209473 | N92.1 | Excessive and frequent menstruation with irregular cycle | Condition | ICD10CM |
| 44827910 | 626.2 | Excessive or frequent menstruation | Condition | ICD9CM |
| **HMB codes in the standardised SNOMED vocabulary as used in OMOP CDM** | | | | |
| 197607 | 266601003 | Excessive and frequent menstruation | Condition | SNOMED |
| 4302555 | 386692008 | Menorrhagia | Condition | SNOMED |
| 196471 | 266602005 | Puberty bleeding | Condition | SNOMED |
| 4028474 | 237125007 | Pubertal menorrhagia | Condition | SNOMED |
| 37311979 | 788919002 | Excessive menstruation with irregular cycle | Condition | SNOMED |
| 4151132 | 314631008 | Menometrorrhagia | Condition | SNOMED |
| 606964 | 1148913001 | Menorrhagia due to bleeding disorder | Condition | SNOMED |
| 4127414 | 237128009 | Metropathia hemorrhagica | Condition | SNOMED |
| 4034020 | 237126008 | Pubertal bleeding and menorrhagia | Condition | SNOMED |

CDM=common data model. HMB=heavy menstrual bleeding. ICD=International Classification of Diseases. OMOP=Observational Medical Outcomes Partnership. SNOMED=Systematized Nomenclature of Medicine.

*Supplementary Table 2:* Overall incidence rates, per 1000 person-years, of heavy menstrual bleeding per data source across all years of observation in the US, UK, and Europe

|  | **CPRD Aurum (n=471 578)** | **CPRD GOLD (n=249 969)** | **IPCI (n=28 109)** | **IQVIA Disease Analyzer^®^ Germany (n=50 190)** | **IMASIS (n=1309)** | **SUCD (n=436)** | **MarketScan (n=2 000 878)** | **Clinformatics^®^ (n=748 066)** | **NLHR@UiO (n=247 383)** |
| --- | --- | --- | --- | --- | --- | --- | --- | --- | --- |
|  | **UK** | **UK** | **Netherlands** | **Germany** | **Spain** | **Hungary** | **USA** | **USA** | **Norway** |
| **All age categories, IR (95% CI)** | 8·74 (8·71, 8·76) | 9·99 (9·95, 10·02) | 8·67 (8·57, 8·78) | 11·70 (11·60, 11·80) | 1·51 (1·43, 1·60) | 0·65 (0·59, 0·72) | 24·09 (24·06, 24·13) | 25·87 (25·82, 25·93) | 13·67 (13·62, 13·72) |
| **Age 11–29 years, IR (95% CI)** | 5·98 (5·95, 6·01) | 6·93 (6·88, 6·99) | 6·94 (6·79, 7·09) | 10·48 (10·30, 10·66) | 1·04 (0·91, 1·18) | 0·26 (0·19, 0·34) | 16·22 (16·18, 16·27) | 18·51 (18·44, 18·59) | 9·57 (9·50, 9·63) |
| **Age 30–44 years, IR (95% CI)** | 9·58 (9·54, 9·62) | 11·53 (11·46, 11·60) | 9·95 (9·77, 10.15) | 14·88 (14·69, 15·07) | 1·67 (1·45, 1·81) | 0·80 (0·70, 0·90) | 32·04 (31·97, 32·11) | 32·70 (32·59, 32·81) | 16·26 (16·16, 16·36) |
| **Age 45–55 years, IR (95% CI)** | 12·38 (12·32, 12·45) | 12·47 (12·38, 12·56) | 9·63 (9·43, 9·83) | 9·60 (9·44, 9·76) | 1·88 (1·71, 2·05) | 0·91 (0·77, 1·06) | 26·48 (26·41, 26·55) | 28·02 (27·89, 28·14) | 17·99 (17·86, 18·13) |

CPRD=Clinical Practice Research Datalink. CI=Confidence Interval. IPCI=Integrated Primary Care Information. IMASIS=Institut Municipal d’Assistència Sanitària Information System. IR = incidence rate. NLHR@UiO=Norwegian Linked Health Registry at University of Oslo. SUCD=Semmelweis University Clinical Data.

Supplementary figures

*Supplementary Figure 1:* Study design

HMB=heavy menstrual bleeding.

*Supplementary Figure 2:* Patient attrition (number of patients per selection stage) for each data source

CPRD=Clinical Practice Research Datalink. HMB=heavy menstrual bleeding. IPCI=Integrated Primary Care Information. IMASIS=Institut Municipal d’Assistència Sanitària Information System. NLHR@UiO,=Norwegian Linked Health Registry at University of Oslo. SUCD=Semmelweis University Clinical Data.

*Supplementary Figure 3:* Annual incidence rates, per 1000 person-years, of heavy menstrual bleeding for each data source

Incidence rate per 1000 person-years

| Year | Clinformatics® | MarketScan | NLHR@UiO | IQVIA Disease Analyzer® Germany | CPRD GOLD | CPRD Aurum | IPCI | IMASIS | SUCD |
| --- | --- | --- | --- | --- | --- | --- | --- | --- | --- |
| 2000 |  |  |  |  | 14.42 | 8.35 |  | 0.94 |  |
| 2001 |  |  |  |  | 11.84 | 7.02 |  | 0.57 |  |
| 2002 |  |  |  |  | 10.84 | 7.24 |  | 0.21 |  |
| 2003 |  | 21.99 |  |  | 11.42 | 8.03 |  | 0.25 |  |
| 2004 |  | 18.39 |  |  | 11.08 | 8.47 |  | 0.28 |  |
| 2005 |  | 18.52 |  |  | 10.12 | 8.38 |  | 0.27 |  |
| 2006 |  | 18.83 |  |  | 10.14 | 8.54 |  | 0.13 |  |
| 2007 |  | 20.48 |  |  | 10.82 | 8.83 | 15.24 | 0.19 |  |
| 2008 | 29.03 | 20.84 |  |  | 10.99 | 8.76 | 11.17 | 0.23 |  |
| 2009 | 24.05 | 24.45 |  |  | 10.89 | 8.93 | 9.61 | 0.16 |  |
| 2010 | 23.6 | 23.34 | 13.93 |  | 10.91 | 8.92 | 8.96 | 0.26 |  |
| 2011 | 23.31 | 22.52 | 12.67 |  | 11.22 | 9.34 | 9.31 | 0.39 |  |
| 2012 | 22.14 | 22.61 | 11.55 |  | 10.62 | 8.96 | 8.65 | 0.43 | 1.63 |
| 2013 | 21.38 | 20.62 | 11.63 |  | 10.21 | 8.91 | 9.45 | 0.5 | 0.54 |
| 2014 | 20.71 | 22.01 | 12.09 |  | 10.05 | 9.18 | 8.76 | 0.62 | 0.86 |
| 2015 | 23.27 | 24.36 | 11.92 |  | 9.48 | 8.94 | 8.43 | 0.65 | 0.88 |
| 2016 | 30.78 | 31.22 | 11.78 |  | 8.74 | 9.01 | 8.85 | 1.03 | 0.73 |
| 2017 | 29.3 | 29.74 | 12.39 |  | 8.44 | 9.1 | 8.52 | 1.23 | 0.65 |
| 2018 | 29.71 | 30.26 | 12.64 |  | 8.25 | 9.42 | 8.75 | 6.77 | 0.43 |
| 2019 | 29.57 | 30.47 | 13.03 |  | 8.46 | 10.14 | 8.67 | 5.68 | 0.4 |
| 2020 | 25.38 | 25.69 | 13.32 | 18.21 | 4.78 | 7.88 | 7.43 | 3.72 | 0.44 |
| 2021 | 28.24 | 28.87 | 15.07 | 10.27 | 5.23 | 8.83 | 8.5 | 4.54 | 0.81 |
| 2022 | 28.23 | 29.57 | 17.13 | 8.96 | 6.03 |  | 8.79 | 7.71 |  |
| 2023 |  |  | 18.72 | 12.15 |  |  | 8.88 |  |  |

CPRD=Clinical Practice Research Datalink. IPCI=Integrated Primary Care Information. IMASIS=Institut Municipal d’Assistència Sanitària Information System. NLHR@UiO=Norwegian Linked Health Registry at University of Oslo. SUCD=Semmelweis University Clinical Data.

***Supplementary Figure 4:* Annual incidence rates, per 1000 person-years, of heavy menstrual bleeding for each data source among women**

**A) aged 11 to 29 years, B) aged 30 to 44 years, and C) aged 45 to 55 years**

*A) 11 to 29 years*

Incidence rate per 1000 person-years

| Year | Clinformatics® | MarketScan | NLHR@UiO | IQVIA Disease Analyzer® Germany | CPRD GOLD | CPRD Aurum | IPCI | IMASIS | SUCD |
| --- | --- | --- | --- | --- | --- | --- | --- | --- | --- |
| 2000 |  |  |  |  | 6.69 | 2.95 |  | 0.49 |  |
| 2001 |  |  |  |  | 5.36 | 2.54 |  | 0.26 |  |
| 2002 |  |  |  |  | 4.98 | 2.91 |  | 0 |  |
| 2003 |  | 10.84 |  |  | 5.5 | 3.17 |  | 0.09 |  |
| 2004 |  | 8.83 |  |  | 5.55 | 3.44 |  | 0 |  |
| 2005 |  | 8.69 |  |  | 5.05 | 3.6 |  | 0.43 |  |
| 2006 |  | 8.75 |  |  | 5.46 | 3.82 |  | 0 |  |
| 2007 |  | 9.94 |  |  | 6.14 | 4.33 | 7.44 | 0 |  |
| 2008 | 15.43 | 10.58 |  |  | 6.32 | 4.38 | 7.78 | 0.08 |  |
| 2009 | 12.69 | 12.57 |  |  | 6.64 | 4.75 | 5.67 | 0.17 |  |
| 2010 | 13.12 | 12.6 | 4.77 |  | 7.3 | 5.06 | 5.42 | 0 |  |
| 2011 | 13.59 | 12.66 | 4.83 |  | 8.27 | 5.81 | 6.27 | 0.35 |  |
| 2012 | 13.37 | 13.53 | 5.01 |  | 8.07 | 6 | 5.74 | 0.36 | 0.17 |
| 2013 | 13.18 | 12.51 | 5.82 |  | 8.12 | 6.22 | 6.86 | 0.37 | 0.27 |
| 2014 | 13.45 | 14.42 | 6.78 |  | 8.7 | 6.79 | 6.76 | 0.39 | 0.25 |
| 2015 | 15.89 | 17.1 | 7.29 |  | 8.1 | 7 | 6.41 | 0.91 | 0.26 |
| 2016 | 22.68 | 23.48 | 7.76 |  | 7.93 | 7.63 | 6.67 | 0.63 | 0.39 |
| 2017 | 22.83 | 23.52 | 9 |  | 8 | 8.37 | 6.25 | 0.88 | 0.08 |
| 2018 | 24.36 | 25.01 | 10.26 |  | 8.34 | 9.06 | 7.16 | 6.01 | 0.08 |
| 2019 | 25.29 | 25.99 | 11.12 |  | 9.1 | 10.27 | 6.94 | 3.58 | 0.17 |
| 2020 | 23.26 | 23.48 | 12.39 | 16.82 | 5.35 | 8.82 | 6.54 | 2.94 | 0.4 |
| 2021 | 24.75 | 25.55 | 14.83 | 8.95 | 5.77 | 9.87 | 7.79 | 2.94 | 0.83 |
| 2022 | 25.13 | 26.17 | 17.5 | 7.93 | 7.52 |  | 8.03 | 7.9 |  |
| 2023 |  |  | 18.59 | 11.09 |  |  | 8.21 |  |  |

*B) 30 to 44 years*

Incidence rate per 1000 person-years

| Year | Clinformatics® | MarketScan | NLHR@UiO | IQVIA Disease Analyzer® Germany | CPRD GOLD | CPRD Aurum | IPCI | IMASIS | SUCD |
| --- | --- | --- | --- | --- | --- | --- | --- | --- | --- |
| 2000 |  |  |  |  | 18.76 | 10.87 |  | 1.71 |  |
| 2001 |  |  |  |  | 15.65 | 9.04 |  | 0.72 |  |
| 2002 |  |  |  |  | 14.13 | 9.19 |  | 0.29 |  |
| 2003 |  | 30.4 |  |  | 14.52 | 10.05 |  | 0.41 |  |
| 2004 |  | 25.39 |  |  | 14.14 | 10.66 |  | 0.37 |  |
| 2005 |  | 25.75 |  |  | 12.86 | 10.39 |  | 0.27 |  |
| 2006 |  | 26.95 |  |  | 12.73 | 10.47 |  | 0.19 |  |
| 2007 |  | 29.37 |  |  | 13.24 | 10.61 | 15.16 | 0.24 |  |
| 2008 | 39 | 29.8 |  |  | 13.2 | 10.37 | 11.33 | 0.22 |  |
| 2009 | 32.75 | 34.62 |  |  | 12.91 | 10.38 | 10.92 | 0.16 |  |
| 2010 | 31.98 | 32.58 | 19.42 |  | 12.6 | 10.18 | 9.71 | 0.21 |  |
| 2011 | 31.41 | 31.1 | 17.42 |  | 12.34 | 10.32 | 11.12 | 0.36 |  |
| 2012 | 29.83 | 31.41 | 15.91 |  | 11.52 | 9.54 | 9.89 | 0.51 | 2.13 |
| 2013 | 28.67 | 28.62 | 15.31 |  | 10.63 | 9.3 | 11.16 | 0.67 | 0.73 |
| 2014 | 27.33 | 29.97 | 15.46 |  | 10.22 | 9.46 | 10.1 | 0.57 | 0.95 |
| 2015 | 30 | 32.7 | 14.91 |  | 9.93 | 9.1 | 9.39 | 0.53 | 1.2 |
| 2016 | 38.61 | 40.38 | 14.23 |  | 8.77 | 8.71 | 10.51 | 1.37 | 0.77 |
| 2017 | 35.96 | 37.37 | 14.46 |  | 8.29 | 8.59 | 10.19 | 1.38 | 1 |
| 2018 | 35.75 | 37.21 | 13.94 |  | 7.86 | 8.86 | 10.13 | 7.25 | 0.5 |
| 2019 | 34.53 | 36.38 | 14.1 |  | 7.94 | 9.36 | 9.88 | 6.61 | 0.51 |
| 2020 | 28.72 | 29.6 | 14.04 | 22.63 | 4.5 | 7.18 | 8.57 | 4.24 | 0.35 |
| 2021 | 32.45 | 33.6 | 15.02 | 13.1 | 5.12 | 8.02 | 9.51 | 4.79 | 0.68 |
| 2022 | 32.78 | 34.95 | 16.89 | 11.35 | 5.22 |  | 10.02 | 8.48 |  |
| 2023 |  |  | 19.08 | 15.7 |  |  | 9.76 |  |  |

*C) 45 to 55 years*

Incidence rate per 1000 person-years

| Year | Clinformatics® | MarketScan | NLHR@UiO | IQVIA Disease Analyzer® Germany | CPRD GOLD | CPRD Aurum | IPCI | IMASIS | SUCD |
| --- | --- | --- | --- | --- | --- | --- | --- | --- | --- |
| 2000 |  |  |  |  | 18.06 | 12.23 |  | 0.5 |  |
| 2001 |  |  |  |  | 14.64 | 10.41 |  | 0.75 |  |
| 2002 |  |  |  |  | 13.79 | 10.66 |  | 0.38 |  |
| 2003 |  | 24.04 |  |  | 15 | 12.45 |  | 0.22 |  |
| 2004 |  | 21.12 |  |  | 14.33 | 13.11 |  | 0.52 |  |
| 2005 |  | 21.78 |  |  | 13.41 | 13.19 |  | 0.1 |  |
| 2006 |  | 22.22 |  |  | 13.27 | 13.53 |  | 0.18 |  |
| 2007 |  | 23.88 |  |  | 14.44 | 13.86 | 24 | 0.35 |  |
| 2008 | 32.3 | 24.21 |  |  | 15.05 | 13.95 | 14.87 | 0.41 |  |
| 2009 | 28.1 | 28.4 |  |  | 14.71 | 14.1 | 12.7 | 0.16 |  |
| 2010 | 27.39 | 26.99 | 21.57 |  | 14.23 | 13.81 | 12.49 | 0.62 |  |
| 2011 | 27.04 | 26.36 | 19.9 |  | 14.4 | 14.12 | 10.98 | 0.53 |  |
| 2012 | 25.75 | 25.97 | 17.33 |  | 13.52 | 13.44 | 10.87 | 0.44 | 2.5 |
| 2013 | 25.1 | 24.36 | 17.45 |  | 13.04 | 13.3 | 10.74 | 0.44 | 0.52 |
| 2014 | 23.91 | 25.04 | 17.61 |  | 12.1 | 13.27 | 9.82 | 0.95 | 1.47 |
| 2015 | 26.62 | 26.79 | 16.83 |  | 11.13 | 12.48 | 9.99 | 0.73 | 1.07 |
| 2016 | 33.66 | 33.68 | 16.37 |  | 10.09 | 12.26 | 9.9 | 0.97 | 1.11 |
| 2017 | 30.87 | 31.5 | 16.36 |  | 9.48 | 11.5 | 9.73 | 1.44 | 0.73 |
| 2018 | 30.07 | 31.06 | 15.77 |  | 8.82 | 11.27 | 9.52 | 7.59 | 0.75 |
| 2019 | 29.67 | 31.02 | 15.6 |  | 8.35 | 11.45 | 9.83 | 6.69 | 0.52 |
| 2020 | 24.07 | 24.67 | 14.39 | 14.81 | 4.41 | 7.37 | 7.58 | 4.07 | 0.66 |
| 2021 | 27.76 | 28.39 | 16.01 | 8.59 | 4.62 | 8.46 | 8.56 | 5.84 | 1 |
| 2022 | 26.52 | 28.17 | 17.03 | 7.51 | 4.96 |  | 8.59 | 7.3 |  |
| 2023 |  |  | 18.74 | 9.54 |  |  | 8.98 |  |  |

CPRD=Clinical Practice Research Datalink. IPCI=Integrated Primary Care Information. IMASIS=Institut Municipal d'Assistència Sanitària Information System. NLHR@UiO=Norwegian Linked Health Registry at University of Oslo. SUCD=Semmelweis University Clinical Data.

*Supplementary Figure 5:* Prevalence (%) of co-occurring conditions in women with heavy menstrual bleeding 365 days before index date among women

**A) aged 11 to 29 years, B) aged 30 to 44 years, and C) aged 45 to 55 years**

*A) 11 to 29 years*

*B) 30 to 44 years*

*C) 45 to 55 years*

*Categorisation of diagnosed conditions considered likely to underlie HMB was guided by the PALM-COEIN classification system for abnormal uterine bleeding [Munro 2011].

CPRD=Clinical Practice Research Datalink. HMB=heavy menstrual bleeding. IPCI=Integrated Primary Care Information. IMASIS=Institut Municipal d’Assistència Sanitària Information System. NLHR@UiO=Norwegian Linked Health Registry at University of Oslo. NR=not recorded. STD=sexually transmitted disease. SUCD=Semmelweis University Clinical Data.

*Supplementary Figure 6:* Prevalence (%) of co-occurring conditions in women with heavy menstrual bleeding 365 days after index date among women

**A) aged 11 to 29 years, B) aged 30 to 44 years, and C) aged 45 to 55 years**

*A) 11 to 29 years*

*B) 30 to 44 years*

*C) 45 to 55 years*

*Categorisation of diagnosed conditions considered likely to underlie HMB was guided by the PALM-COEIN classification system for abnormal uterine bleeding.^23^ CPRD=Clinical Practice Research Datalink. HMB=heavy menstrual bleeding. IPCI=Integrated Primary Care Information. IMASIS=Institut Municipal d’Assistència Sanitària Information System. NLHR@UiO=Norwegian Linked Health Registry at University of Oslo. NR=not recorded. STD=sexually transmitted disease. SUCD=Semmelweis University Clinical Data.
